# Zinc distribution within breast cancer tissue of different intrinsic subtypes

**DOI:** 10.1007/s00404-020-05789-8

**Published:** 2020-09-15

**Authors:** Peter Rusch, Alfred V. Hirner, Oliver Schmitz, Rainer Kimmig, Oliver Hoffmann, Maxim Diel

**Affiliations:** 1 Applied Analytical Chemistry, Department of Chemistry, University of Duisburg-Essen, Universitätsstraße 5-7, 45141 Essen, Germany; 2 Department of Gynecology and Obstetrics, School of Medicine, University of Duisburg-Essen, Hufelandstr. 55, 45122 Essen, Germany

**Keywords:** Breast cancer, Zinc, Laser ablation inductively coupled mass spectrometry (LA-ICPMS), Steroid receptor, Human epidermal growth factor receptor 2, Biomarker

## Abstract

**Purpose:**

To show feasibility of laser ablation inductively coupled mass spectrometry (LA-ICPMS) for analysis of zinc content and concentration in breast cancer tissue and to correlate this with validated prognostic and predictive markers, i.e. histological grading and expression of steroid receptors (estrogen receptor, ER; progesterone receptor, PR) and human epidermal growth-factor receptor 2 (Her2).

**Methods:**

28 samples of human invasive ductal breast cancer tissue were subclassified into groups of four different intrinsic subtypes according to the expression of ER, PR and Her2 by immunohistological staining and then analyzed for zinc content and distribution by LA-ICPMS applying a calibration technique based on spiked polyacrylamide gels. A correlation of zinc concentration with histological grading and molecular subtypes was analyzed.

**Results:**

Consistent with results of a pilot-study LA-ICPMS was feasible to show zinc accumulation in cancerous tissue, even more adjacent healthy stroma was with proportional increase of zinc. Zinc levels were most elevated in triple-positive (TPBC) and in triple-negative (TNB) breast cancers.

**Conclusion:**

LA-ICPMS was feasible to confirm a connection between zinc and grade of malignancy; furthermore, focusing on a correlation of zinc and intrinsic breast cancer subtypes, LA-ICPMS depicted an upwards trend of zinc for “high-risk-cancers” with highest levels in Her2-positive and in triple-negative (TNBC) disease. The currently uncommon alliance of clinicians and analytical chemists in basic research is most promising to exploit the full potential of diagnostic accuracy in the efforts to solve the enigma of breast cancer initiation and course of disease.

## Introduction

Breast cancer is the most common cancer in women worldwide and still, it is the fifth most common reason for death from cancer in women. In the United States (US), lifetime risk for invasive breast cancer is one in eight [1].

With a peak incidence in postmenopause, an increasing number of premenopausal very young women is affected. About 5–10% of breast cancer is related to gene mutations with a focus on BRCA1- and 2-mutations [2]. A heterogeneous complex of diseases exists with distinct biological features that lead to different treatment options and clinical outcomes.

Still clinical markers commonly used to classify breast cancer are tumor size, lymph node involvement, histological grade, expression of steroid receptors (estrogen receptors [ER]/progesterone receptors [PR]) and human epidermal growth factor receptor 2 (Her2), but recent studies have focused on more detailed biological characteristics to improve patient risk stratification and the benefit to side-effect ratio from a specific treatment modality [3–5].

In this context, Perou [6] and Sorlie [7] were the first to analyze gene expression patterns in breast cancer and to investigate their clinical relevance. The result was a molecular subclassification of different subtypes of breast cancer characterized by clusters with expression of distinct genes coding for steroid receptors, Her2 protein and the proliferation marker Ki57 [8]. This was only the prologue for an even more sophisticated insight into the complex pathway of tumor signaling.

Association of these “molecular” subtypes with course of disease and outcome [9] revealed, that distinct breast cancers have their own unique “intrinsic” molecular portrait. It was shown that Her2-positive and triple-negative breast cancers (TNBC) generally had the poorest survival [10]. While the introduction of Her2-targeted therapies dramatically improved outcomes for the Her2-positive subgroup [11, 12], TNBC is still with unfavorable prognosis due to the absence of a respective target receptor and is merely treated by chemotherapy.

High-throughput methods for gene expression and subsequent categorization of different breast cancer subtypes is now established in commercial tests already [13–15]. Interestingly, despite differences in candidate genes in each of the assays most of them quite reliably predict the biology (metastases-free-/overall survival) of the tumor tested. While these tests focus on certain but different mutations in driver-genes they all show more or less feasibility as prognostic and/or predictive tool for decision making. In fact, it is possible that—unless the great benefit of molecular testing—the link to solve the enigma between the genetic portrait and the biological behavior of breast cancer is still missing.

The trace metal zinc (Zn) is known to be involved in multiple cellular processes [16]. Being a cofactor for more than 300 enzymes (e.g. all RNA-polymerases are zinc metalloenzymes) zinc contributes to cellular signaling, proliferation, homeostasis, immunofunction, oxidative stress, apoptosis and aging [17, 18]. Zinc-associated proteins are metallothioneins, zinc transporters (esp. ZnT2, and Zip 6, 7, 10), p53 tumor suppressor and matrix metalloproteases—factors all being involved in carcinogenesis and tumor progression. This may be true particularly for breast cancer where zinc is at the interface of checkpoints of cancer initiation, promotion and progression while influencing gene expression at the level of the cell nucleus by stabilizing structure and thus regulating transcription factors, e.g. nuclear factor-kappa B (NF-κB) [19–23].

Intracellular zinc is important for rapidly dividing cancer cells, thus zinc homeostasis in normal cells being dysregulated in cancer cells [21]. Zinc levels in cancerous and in non-cancerous tissues were analyzed in many studies since the 70 s [24–29]. Elevated zinc levels were found in cancers of the breast, the colorectum, and of head and neck, while cancers of other origin (i.e. prostate, liver, stomach) show an inverse correlation [30]. Only one author describes decreased zinc levels in breast cancer [31].

With regard to the relevance of cancer-initiation, Cui observed a correlation between high levels of zinc in breast tissue and the onset of carcinogenesis [32]. Zinc levels were found to be higher in estrogen-receptor (ER)-positive breast cancers [33], while distinctive “subtype”-specific dysregulations of zinc-homeostasis were described [22]. Tamoxifen-resistant breast cancer cells showed with increased intracellular zinc [34]. Again, authors proposed the role of the zinc-transporter ZIP7 in the control of intracellular zinc homeostasis [35].

While the optimal immunostimulatory dose of zinc has not yet been determined, research focuses also on dietary effects and on suitable chemotherapies. In this context, there is evolving evidence for zinc not only as a (diagnostic) biomarker but as a therapeutic agent, too [36–38].

**Fig. 1:**
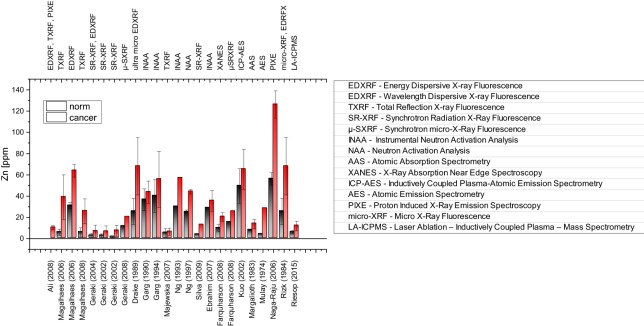
Different analytical methods for zinc level testing in normal and cancer breast tissue; literature overview. (For numerical values see Appendix Table 2)

Besides well-established histopathological analyses, different analytical methods exist for the detection and diagnosis of cancer. Synchrotron micro-X-Rax-Fluorescence (µ-SXRF) or Laser-Ablation-Inductively Coupled Mass Spectrometry (LA-ICPMS) are spacial resolving analytical methods providing a kind of “heatmap” of distribution and concentration of distinct trace elements in a tissue sample. These analytical techniques have shown equivalence in differentiating between cancer cell clusters and adjacent stroma. Analysis was feasible on a single tissue section and was not dependent on paired samples (see Fig. 1).

Discrepancy of zinc level and distribution between normal and cancerous tissue can range widely—from very few to more than 80 ppm. This may correlate with histologic grading and expression of steroid-receptor-status [33].

A feasibility study of LA-ICPMS for zinc analysis in breast cancer showed correlations of zinc amount, concentration, and distribution with histopathological phenotypic grade (G) of malignancy in breast cancer. Zinc levels roughly doubled from G1 to G3 cancer. Nonetheless, this was shown on a small set of nine samples only [39].

This present study was intended as a follow-up study according to Riesop [39], thus using LA-ICPMS on a bigger set of 28 human breast cancer samples. This time zinc content and concentration of LA-ICPMS-analysis were correlated with “molecular/intrinsic” subtypes according to the immunohistochemical expression of steroid (ER/PR) and Her2 receptors.

## Methods

### Tumor tissue samples

A set of 28 samples of breast cancer tissue was carefully selected and divided into a subset of four “intrinsic” subgroups according to their immunohistochemical receptor profile (steroid- (hormonal-) receptor (HR) positive (HRpos.)/Her2neg. vs. HRneg./Her2pos. vs. HRpos./Her2pos.[triple-positive, TPBC] vs. HRneg./Her2neg.[triple-negative, TNBC]). No additional clinical data was available.

The primary outcome was the determination of zinc concentration and distribution via LA-ICPMS-analysis. The second outcome was the correlation of zinc results with the “intrinsic subtypes” according to immunohistochemical receptor status.

Tissue samples were stored at − 85 °C in the tissue bank of University Hospital of Essen and were dissected into 10-µm-thin slides by cryomicrotom. Two subsequent sections were prepared on glass beads. One sample was stained with hematoxylin and eosin (H&E) and used for microscopic histopathological evaluation. The other section was destined for LA-ICPMS analysis in the Institute for Applied Analytical Chemistry of University of Duisburg-Essen. Storage of tumor samples before processing was at − 80 °C.

### Instrumental zinc distribution analysis

Zinc distribution in the tumor samples was analyzed with LA-ICPMS. The system utilized consisted of a Laser Ablation System UP 213 FP (New Wave Research, ESI Inc., Fremont, CA, USA) and an ICPMS 7500a (Agilent Technologies, Yokogawa, Japan). Transfer of tumor samples to the Institute of Applied Analytical Chemistry was on an aluminum block cooled by liquid nitrogen which was then positioned in a cryoablation chamber held at − 15 °C and which was purged with helium for elimination of oxygen and water.

Zinc quantification in the tumor samples was performed by application of the method of matrix adapted calibration using a 12.5% polyacrylamide gel spiked with Zn standard solution before polymerization; this procedure had already been validated in the course of a PhD thesis and was successfully applied by Riesop [39].

To exclude particulate contaminations, all manipulations were performed within a flow-box.

The laser ablation cell and all related tubings were purged with helium. The LA-ICPMS was then tuned for maximal intensity using NIST SRM 612; important measuring parameters are listed in Table 1.

**Table 1:** Applied LA-ICPMS parameters according to Riesop et al. [39]

Instrumental parameters (unit)	Values
Rf power (W)	1550
Flow rate plasma gas (L/min, Ar)	15
Flow rate carrier gas (L/min) (Ar)	1.1–1.2
Scan modus	Peak hopping
Sampling time m/Z (s)	0.1
Laser spot size (μm)	200
Scan speed (μm/s)	50
Isotopes monitored	13C, 31P, 34S, 54Fe, 57Fe, 63Cu, 65Cu, 64Zn, 66Zn, 129Xe
Flow rate ablation gas (L/min) (He)	1.2–1.3
Frequency (Hz)	20
Energy density (mJ/cm^2^)	0.8
Laser warm-up time (s)	5
Washout time (s)	20

With a laser spot size of 200 µm, an ablation line overlapping at 20 µm, an ablation speed of 50 µm/s, and an ICP-MS sampling time of 1 s the final image resolution was 180 × 50 µm^2^.

The elemental contour plots were not smoothed and show element distribution after subtraction of analytical blank. A relatively high sampling inhomogeneity for solid aerosol injection compared to a liquid sampling resulted in a relative standard deviation of approximately 17% for all measurements.

**Fig. 2:**
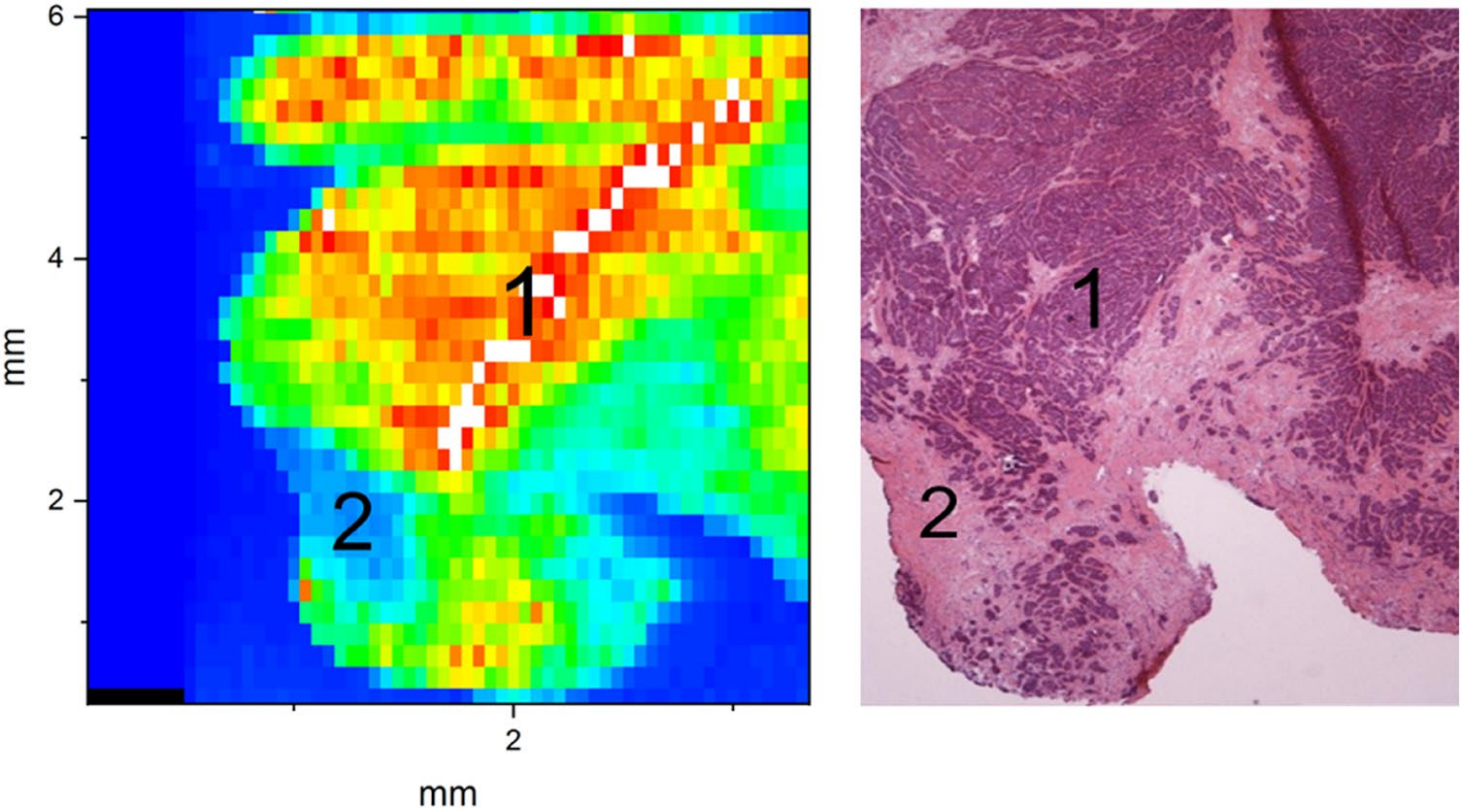
“Heatmap” of zinc distribution and quantification by LA-ICPMS (left) and by histological H&E-staining (right) of parallel sections in a representative tumor sample. 1—Malign area, 2—stroma area

Cooperation with local pathologists vouched for accuracy of allocation of stromal and cancerous areas in the tumor sample and alignment of section plane in hematoxylin and eosin (H&E) staining with section plane in zinc-ablation. An example of zinc distribution and quantification in LA-ICPMS and in histological H&E staining is shown in Fig. 2. The relevant data of sample classification is shown in the “Appendix” (Table 3).

## Results

LA-ICPMS-guided determination of cancerous vs. non-cancerous stromal areas was successful in 26 of 28 samples. Two samples were censored as they were inappropriate for lateral resolution by LA-ICPMS, because the portion of healthy stroma vs. cancerous tissue was too small. Table 3 (see “Appendix”) shows numerical results of zinc analysis in the individual tumor sample.

Zinc concentration ranged from 0.8 to 11.4 ppm for stromal and from 3.5 to 19.5 ppm for cancer areas, respectively.

**Fig. 3:**
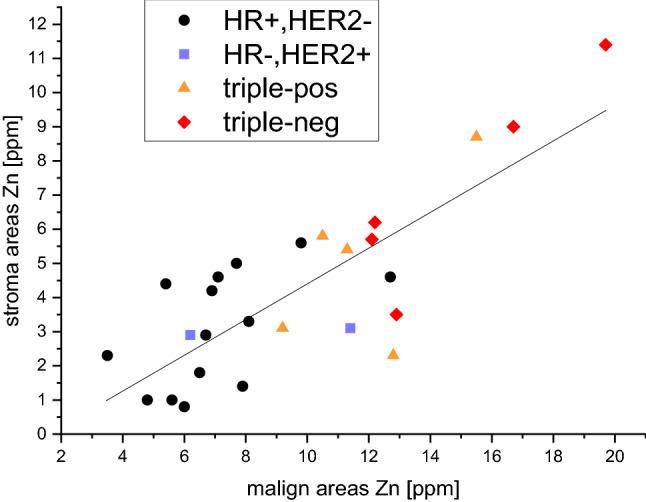
Comparison of Zinc(stroma)/Zinc(cancer)-ratio of cancer fields vs. (benign) adjacent stroma in different “intrinsic” breast cancer subtypes with a constant ratio over a broad concentration range. (The data set passed the Kolmogorov–Smirnov test for normal distribution.)

While zinc quantification showed a definite increase in malign areas of the tumor sample, this was paralleled by a mild increase in (benign) adjacent stroma, leading to a relatively constant Zn(stroma)/Zn(cancer) ratio of 2.9 ± 1.6 (Fig. 3).

**Fig. 4:**
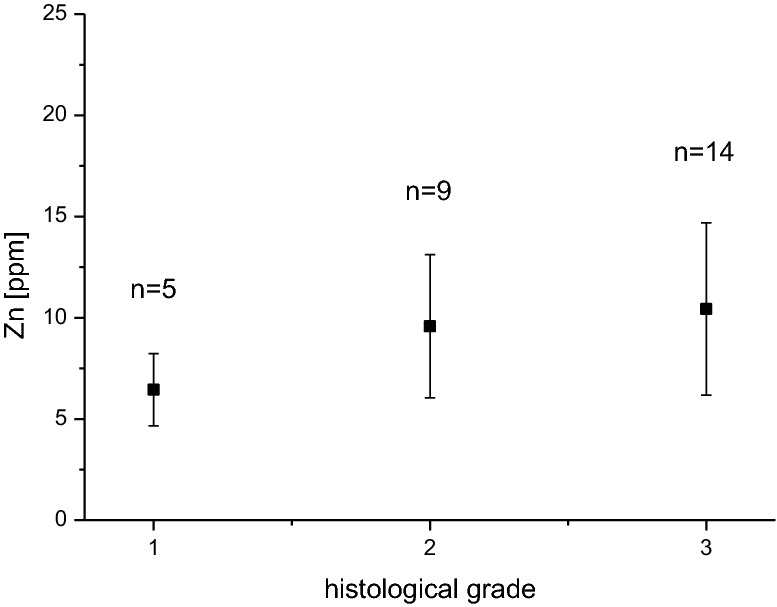
Error bar plot of zinc level and histopathological grade of malignancy

In line with a pilot-study [39], a trend of zinc level rising with the higher histopathological grade of malignancy (G1–3) was confirmed (Fig. 4). However, statistical significance could not be proved.

**Fig. 5:**
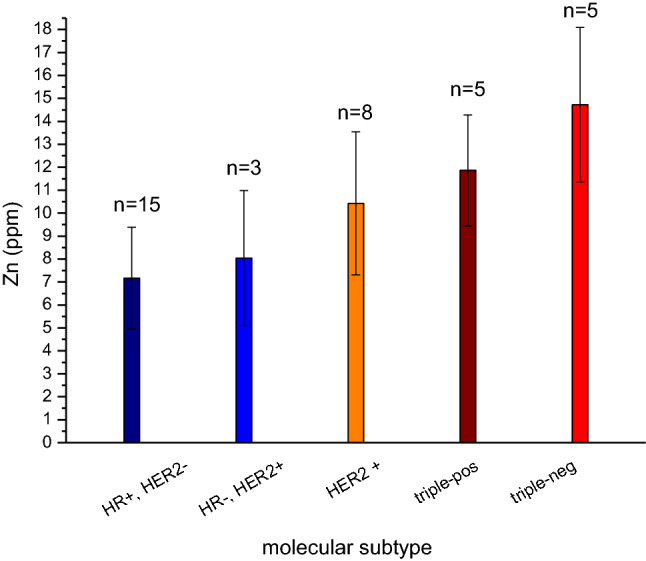
Error bar plot of zinc-level and intrinsic subtype according to their distinct receptor-profile (according to [40])

A relevant trend of zinc elevation for intrinsic subtypes was observed towards Her2-positive- and towards triple-negative breast cancers. While breast cancers with only positive steroid receptors showed just mild zinc elevations, zinc increased with the expression of the Her2 receptor, thus being higher in HRneg/Her2-positive and even higher in triple-positive (HRpos/Her2pos) breast cancers. Finally, triple-negative (HRneg/Her2neg) breast cancers presented with the highest zinc results (Fig. 5).

**Fig. 6:**
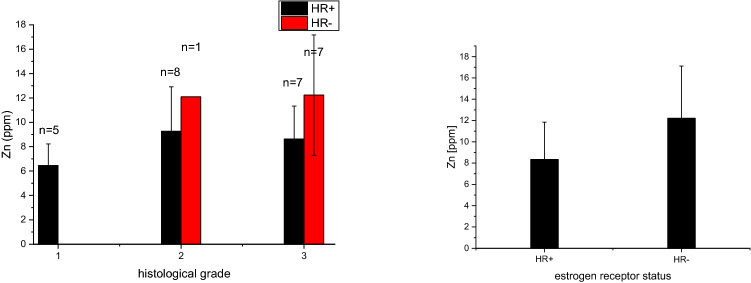
**a** Error bar plot of zinc and grading within the HR + /HR- subgroups; **b** Error bar plot of zinc-level and expression of steroid-receptors

Due to size of the sample subsets, a correlation of zinc levels with histological grading according to steroid-receptor-status was only applicable for grade 2 and 3 with a clear trend with highest zinc levels in the HR-negative subgroups (Fig. 6a), but statistical significance could not be proved. Focusing on the steroid-receptor-status only—irrespective of the grade of malignancy—the zinc increase was even more evident in the HR-negative subgroup. It was 68% higher in HR-negative than in HR-positive breast cancer samples (Fig. 6b).

## Discussion

With regard to the prevalence of zinc in multiple cellular processes and its relevance in cancer this study aimed at these distinct goals:

1. To proof feasibility of LA-ICPMS as a prototype for zinc-analysis in breast cancer; a pilot study demonstrated general applicability of LA-ICPMS for zinc-analysis in breast cancer, but this was only on a very small set of samples [39]. This study was conducted as the confirmatory follow-up study.
2. To relate zinc to breast cancer subtypes according to a distinct pattern of receptor expression.

Status of steroid receptor- and Her2-status expression are the most relevant prognostic and predictive factors routinely determined in histopathology of breast cancer [8, 41, 42]. Genetic profiling allows subgrouping breast cancer into distinct subtypes with subgroup-specific biological behavior [6–8, 42–44].

Different definitions exist to classify breast cancer into “molecular” or “intrinsic” subtypes. Most commonly distinct patterns of receptor expression and presence of the proliferation marker Ki67 are used for subgrouping. As no clear cut-off-values exist for the definition of Ki67 being “high” or “low”, our analysis focused on a simplified classification of molecular subtypes with the focus on receptor expression only and with omission of Ki67 [6, 7]. One goal of this study was to relate the results to the expression of the aforementioned receptors.

LA-ICPMS was successfully applied to zinc analysis on a set of 28 breast cancer samples. Zinc was found to be 68% higher in HRneg than in HRpos samples; this follow-up-study confirmed most evident zinc elevations in grade 3 (G3) breast cancers.

At the same time zinc was related to the respective breast cancer-subgroup according to the expression of aforementioned receptors with a continuum of zinc-increase from HRpos to Her2pos and finally TPBC (HRpos/Her2pos) and TNBC (HRneg/Her2neg). Unfortunately, due to size of the sample set and as no paired samples where available for analysis, it was impossible to determine if the zinc elevation contributed mainly to the negative steroid-receptor or to the higher grading.

A correlation of the expression of steroid-receptors and histological grade of malignancy was shown by Badowska: Among 231 breast cancer cases, the incidence of positive steroid receptors was highest in the intermediate-risk (G2) subgroup and lowest in the high-risk (G3) subgroup [45].

Results of our own study showed a relation of zinc with steroid receptor profile and with histological grading. No literature exists to answer if zinc concentration according to intrinsic subtypes superimposes that according to histological grading.

Rare studies investigate the role of zinc in the context of different intrinsic breast cancer subtypes: Chandler [22] analyzed the subtype-specific accumulation of zinc in a small study of only six tumor samples with only two different receptor profiles (TPBC, TNBC, non-malignant reference). While using a less precise XRF-microprobe with a lower spatial resolution than in LA-ICPMS, he concludes that intracellular “zinc-management may underlie phenotypic characteristics of breast cancer such as grade, invasiveness, metastatic potential and response to therapy”.

Applied analytical research is feasible to trace zinc to its regulatory destination and to visualize zinc-dependent checkpoints for cancer spread. Kim [46] and Chandler [22] used the zinc-responsive fluorophore FluoZin-3 for a correlation of zinc and distinct zinc-transporters in different intrinsic subtypes. They demonstrated different zinc-levels in steroid-positive compared to steroid-negative (basal-like) cancers. They hypothesized that the zinc-transporting network may play a key role in distinct subtype-specific biological aggressiveness.

Unlike our own results, Costello [31] observed a downward trend for zinc concentration with higher histological grade, but analysis was based on semi-quantitative zinc determination. Farquharson [33] has shown an overall 60% higher zinc level in the ER + than in the ER-, however, only grade 1 samples were comprised.

Hypotheses exist with regard to a stepwise development of breast cancer. Zinc appears elevated in breast cancer compared to healthy breast tissue [26, 27]. Nandi found that zinc was 19-fold higher in cancer areas than in the healthy counterpart [47]. In this context, the role of the (healthy?) adjacent stroma as a promoting tumor microenvironment is currently under intensive investigation [48, 49].

Our own study showed a relevant increase of zinc in cancer areas that was paralleled by a mild zinc elevation in adjacent stroma, leading to a relatively constant Zn(stroma)/Zn(cancer) ratio of 2.9 ± 1.6. This result is in line with existing literature with values ranging between 1.2 [50] and 6.5 [51].

Hypothetically, stroma-zinc represents the background-risk of breast cancer. In this context, publications discuss the relevance of tumor-stroma as tumor microenvironment promoting cancer growth and initiation of metastasis [52], e.g. via estrogen-receptor-activation [53], via direct or indirect interactions of zinc with the immunosystem [54], via crosstalk between immune cells and cancer cells [55] or via zinc-mediated signaling in growth factor activation [53, 56].

In contrast to the aforementioned literature, again only Costello [31] observed an inverse correlation of zinc with cancer fields and adjacent stroma, but this was with an overcome analytical technique and on a small sample size without consideration of receptor expression.

A gross correlation of zinc-content and -distribution with histological grading in breast cancer was already reported in a former pilot study [39]. The confirmatory results of our follow-up-study are with a higher statistical standard deviation, which may be due to the higher number of analyzed samples. Additionally, sample selection criteria for this study were rather based on intrinsic subtypes than on grading.

In our work, zinc levels and intrinsic breast cancer subtypes were related to each other with an upwards trend for “high-risk-cancers” [10, 57], while “luminal-like types” (HRpos./Her2neg.) had only mild zinc increases, zinc levels were considerably higher in Her2-positive and finally being highest in triple-negative breast cancers (TNBC). Hypothetically zinc level reflects the continuum from “low-risk” steroid-positive (luminal) breast cancers (with options for antitumor treatment via the respective target-receptor) via “intermediate-risk” Her2-positive breast cancers (with options for Her2-targeted therapy) to finally autonomous TNBC without any targets for cancer regulation.

Tamoxifen is a selective estrogen-receptor modulator with anti-cancer effects on steroid-receptor-positive breast cancer. Over time of disease a loss of Tamoxifen effect is possible [58]. In this context, Taylor analyzed zinc with regard to developing Tamoxifen resistance via the respective steroid-receptor [56]. The zinc transporter ZIP7 was increased in Tamoxifen-resistant (TamR) breast cancer cells, resulting in a zinc-wave triggering tumor growth via the activation of growth factor-receptors. Provided that—from a biological standpoint—TamR-cells are comparable to HRneg-cells, a suchlike zinc-wave may be the surrogate for our findings of higher zinc levels in HRneg than in HRpos breast cancers.

Accordingly, Lopez and Kelleher [59] showed that overexpression of ZIP6 (LIV-1) zinc-transport-protein is common in ER-positive breast cancer and results in higher intracellular zinc-levels compared to normal breast cells. Conversely, ZIP6-attenuation significantly reduced cellular zinc pools which resulted in decreased apoptosis and finally reduced E-cadherin-expression, which is regarded as a key step in the process of epidermal–mesenchymal transition initiating metastasis.

Analytical research in the context of breast cancer is currently uncommon compared to well-established histopathological analysis. In the context of zinc and breast cancer results of analytical research are often impaired by the use of insufficiently characterized tumor samples not suitable for correlation with histopathological data. Only 1 of 22 publications (see Fig. 1) worked on samples classified according to histopathological grading and clinical stage. A correlation of zinc and estrogen-receptor status was only analyzed by Farquharson [33] and Riesop [39].

Application of LA-ICPMS in clinical routine is limited due to complexity: zinc analysis of a tissue section of approximately 1 cm^2^ costs several hours, while the test setup and the data evaluation are complex. Finally, it is destructive by vaporization of the analyzed tissue samples, so reproducibility of results is limited.

Yet, analytical research with spatial ablation prepares the ground for advantageous alternative techniques. Cortesi demonstrated feasibility of a non-destructive X-ray-fluorescence for zinc-analysis in screening for prostate cancer [60].

The present study confirms results of a former pilot study with regard to a relation of zinc and histopathological grading in breast cancer. Additionally, this work highlights an association of zinc level with “intrinsic” subtypes in breast cancer according to the expression of steroid- and Her2-receptors. Provided that steroid- and Her2-receptors are of predictive and of prognostic value [3, 61] in breast cancer, zinc level may be a surrogate for the inherited risk of the disease.

Still, the core feature of zinc in the context of breast cancer has to be defined.

## Conclusion

There is growing evidence that zinc-homeostasis is a keystone in health and imbalance contributes to cancer initiation and progression.

28 samples of breast cancer tissue were analyzed for zinc content with LA-ICPMS. Results of a pilot study were confirmed and showed elevated zinc levels with increase of the histopathological grade of malignancy.

Relevant zinc increase in cancer areas of the sample were paralleled by mild increases in the adjacent (healthy) stroma, resulting in a relatively constant Zn(stroma)/Zn (cancer) ratio (2.9 ± 1.6). This may hint at the surrounding stromal tissue as “tumor-microenvironment” being involved in the course of disease (initiation, progression).

Zinc analysis according to four different “intrinsic” breast cancer subtypes showed a relation of zinc-content with the expression of steroid and Her2 receptors. The connection was closest for “high-risk” cancers, i.e. triple-positive and triple-negative breast cancers. As the latter were mainly of histological grade 3 at the same time, it was not possible to determinate if receptor or grading count more for zinc-elevation.

HRpos samples showed zinc by 68% higher than HRneg samples. This is in accordance with most of the existing literature.

Some limitations exist in this study: Still, the sample size is small. It was not possible to correlate analytical results to multiple other zinc-associated parameters discussed in literature, i.e. labile zinc (“Zn2 + ”), zinc transporters or metallothioneins. Standard deviations are relatively high, and it is not possible to determine breast cancer classifications based on zinc content only.

LA-ICPMS for zinc analysis does not seem feasible for clinical routine as it is time consuming and complex with regard to the test setting and the data evaluation. Finally, results cannot be reproduced as vaporization destroys the sample but is essential for the technique. Nonetheless, accuracy of well-established histopathological examinations may benefit from applied analytical research: a next step is the development of similar application studies focusing on analytical techniques feasible for the integration into the clinical routine, e.g. X-ray fluorescence analysis [51, 60, 62], may it be as a supplementary working tool for the histopathologist.

Oncological research in a multidisciplinary setting with basic researchers, clinicians and analytical chemists is new. It may contribute new translational attempts to highlight the core function of zinc in breast cancer and may help to solve the enigma of breast cancer genesis and course of disease.

## Appendix

See Tables 2 and 3.

**Table 2:** Zinc (Zn)- level in normal and breast cancer tissue, literature overview

Studies	Author	Comment	*n*	Mean normal (ppm Zn)	STD	*n*	Mean cancer (ppm Zn)	STD	Analytical method
1	Ali (2008) [63]					24	10.2	2.0	EDXRF, TXRF, PIXE
2	Magalhaes et al. [51]		1	6.0	3.0	1	39.0	21.0	TXRF
3	Magalhaes et al. [51]		1	31.0	3.0	1	64.0	6.0	EDXRF
4	Magalhaes et al. [64]		6	6.0	4.3	6	25.8	11.6	TXRF
5	Geraki et al. [65]			2.9	2.0		6.9	5.8	SR-XRF, EDXRF
6	Geraki et al. [66]	Paired	20	2.7	1.8	20	6.5	5.5	SR-XRF
7	Geraki et al. [66]	Independent	20	1.7	0.9	20	7.4	5.2	SR-XRF
8	Geraki et al. [67]	Spacial res	1	10.9	5.6	1	23.9	8.8	µ-SXRF
9	Drake et al. [68]		26	25.6	12.1	26	68.1	26.9	ultra micro-EDXRF
10	Garg et al. [69]		4	36.6	10.2	4	43.8	10.5	INAA
11	Garg et al. [70]		30	40.1	15.3	30	55.8	26.3	INAA
12	Majewska et al. [50]		68	5.4	3.9	26	6.4	3.4	TXRF
13	Ng et al. [71]	Median, paired	15	30.0 (14–42)		15	57.0 (28–113)		INAA
14	Ng et al. [72]		46	25.0	1.9	46	44.2	2.1	NAA
15	Silva et al. [73]	Paired	26	3.8		26	12.9		SR-XRF
16	Ebrahim et al. [74]	Dry		28.8			35.5	9.8	INAA
17	Farquharson et al. [75]		9	9.9	3.2	9	20.4	4.0	XANES
18	Farquharson et al. [75]	Spacial res	2	15.4		2	25.5		µSRXRF
19	Kuo et al. [76]	Wet paired	25	49.6	16.1	25	65.2	18.8	ICP-AES
20	Margalioth et al. [26]	Wet	4	8.1	0.6	8	13.9	4.4	AAS
21	Mulay et al. [25]	Dry	8	4.0	(1.1–9.3)	8	28.3	(8.5–84)	AES
22	Naga-Raju et al. [77]	Dry	18	56.2	5.8	18	126.2	12.7	PIXE
23	Rizk et al. [78]	Dry	22	25.6	12.1	22	68.1	26.9	micro-XRF EDRFX
24	Riesop et al. [39]	Wet tissue, special res	9	6.0	1.6	9	12.0	4.6	LA-ICPMS

**Table 3:** Zinc concentrations of “cancer fields” and of “adjacent stroma” in 28 breast cancer tissue sections

	Sample name	Grade	Subgroup	Proportion of cancer [%]	Zn-concentration “cancer fields” [ppm]	Zn-concentration “adjacent stroma” [ppm]
1	E171-07	1	HRposHER2neg	20	6.0 ± 1.2	0.8 ± 0.2
2	E30921-10	1	HRposHER2neg	30	7.7 ± 1.6	5.0 ± 0.6
3	E15529-9	1	HRposHER2neg	40	3.5 ± 0.4	2.3 ± 0.4
4	E16137-09	1	HRposHER2neg	40	7.9 ± 2.6	1.4 ± 0.2
5	E16883-08	1	HRposHER2neg	80	7.1 ± 0.6	4.6 ± 0.6
6	122-536I	2	triple-neg	60	12.1 ± 3.2	5.7 ± 1.2
7	226-999III	2	HRposHER2pos	60	11.3 ± 0.8	5.4 ± 0.7
8	193-902IV	2	HRposHER2pos	90	15.5 ± 1.4	8.7 ± 0.7
9	171-357I	2	HRposHER2pos	80	12.8 ± 1	2.3 ± 0.4
10	E17591-07	2	HRposHER2neg	50	8.1 ± 0.8	3.3 ± 0.6
11	E28891-06	2	HRposHER2neg	75	5.4 ± 0.6	4.4 ± 0.6
12	E30167-08	2	HRposHER2neg	90	8.9 ± 1.0	–
13	E13244-8	2	HRposHER2neg	40	6.5 ± 1.0	1.8 ± 0.4
14	E25812-07	2	HRposHER2neg	85	5.6 ± 0.6	1 ± 0.2
15	185-800III	3	HRposHER2pos	70	9.2 ± 0.8	3.1 ± 1.0
16	187-827III	3	HRposHER2pos	90	10.5 ± 1	5.8 ± 1.3
17	E16996-8	3	HRnegHER2pos	40	11.4 ± 1.5	3.1 ± 0.6
18	E16452-10	3	HRnegHER2pos	95	6.5 ± 0.6	-
19	E15334-8	3	HRnegHER2pos	25	6.2 ± 0.6	2.9 ± 0.2
20	E30802-07	3	HRposHER2neg	75	6.7 ± 1.0	2.9 ± 0.4
21	E6408-08	3	HRposHER2neg	90	9.8 ± 1.0	5.6 ± 1.0
22	E2141-07	3	HRposHER2neg	90	6.9 ± 0.6	4.2 ± 0.8
23	E25767-06	3	HRposHER2neg	10	4.8 ± 1.2	1.0 ± 0.2
24	E5809-08	3	HRposHER2neg	50	12.7 ± 1.8	4.6 ± 0.8
25	17-1344 V	3	Triple-neg	95	12.9 ± 1.3	3.5 ± 1.3
26	176-734III	3	Triple-neg	90	12.2 ± 2.3	6.2 ± 1.0
27	322-124IV	3	Triple-neg	90	16.7 ± 2.6	9 ± 1.9
28	112-475II	3	Triple-neg	70	19.7 ± 2.7	11.4 ± 2
